# Editorial: Nutritional strategies and diet-microbiota interaction to improve skeletal muscle function

**DOI:** 10.3389/fnut.2025.1579437

**Published:** 2025-03-10

**Authors:** Guang-xu Ren, Li-jing Ke

**Affiliations:** ^1^Institute of Food and Nutrition Development, Ministry of Agriculture and Rural Affairs of the People's Republic of China, Beijing, China; ^2^School of Food Science and Nutrition, University of Leeds, Leeds, United Kingdom

**Keywords:** nutrition, muscle function, gut microbiota, nutrients, muscle atrophy

In this Research Topic, several studies have examined the relationship between nutritional strategies, diet-microbiota interactions, and skeletal muscle function, particularly in the context of skeletal muscle atrophy. These studies have investigated different dietary and metabolic factors that influence muscle health and provide valuable insights into potential interventions to improve skeletal muscle function and prevent muscle loss.

With respect to oxidative balance and skeletal muscle health, Zhao et al. investigated the Oxidative Balance Score (OBS) using data from NHANES (2011–2018). Their findings demonstrated that individuals with a higher OBS had greater skeletal muscle mass and strength, indicating that antioxidant-rich diets and healthy lifestyle choices may help protect against sarcopenia by mitigating oxidative stress.

Regarding micronutrition intake and muscle function, Li et al. explored the association between dietary selenium intake and sarcopenia in American adults. Their results showed an inverse correlation between selenium consumption and sarcopenia prevalence, with higher selenium intake linked to better muscle mass and function. These findings highlight the role of selenium in maintaining skeletal muscle health.

On the Research Topic of gut microbiota and metabolic health, Myhrstad et al. examined the relationship between gut microbiota composition, metabolic markers, and physical activity in healthy adults. Their study found that specific gut bacteria were associated with improved metabolic regulation and muscle performance, reinforcing the potential of gut microbiota modulation in muscle health.

Runting et al. analyzed the link between dietary patterns and bone mineral density (BMD) in middle-aged and elderly individuals. The authors found that diets rich in choline, selenium, and protein were correlated with higher BMD, suggesting that nutrient-dense dietary approaches can support both bone and muscle health.

Finally, Liu et al. explored the role of dietary omega-3 fatty acids in the prevention of osteoporosis. Their study demonstrated a significant inverse relationship between omega-3 intake and osteoporosis risk.

Collectively, the studies in this Research Topic emphasize the multifaceted role of nutrition, oxidative stress, and gut microbiota in skeletal muscle function. Key takeaways include:

- Antioxidant-rich diets may help mitigate oxidative stress and muscle atrophy.
- Selenium intake is associated with reduced risk of sarcopenia and improved muscle mass.
- Gut microbiota composition plays a role in metabolic regulation and muscle function, highlighting the potential for microbiota-targeted interventions.
- Balanced dietary patterns, rich in essential nutrients like choline, selenium, and protein, support both bone and muscle health.

These findings suggest that precision nutrition approaches that integrate gut microbiota modulation, antioxidant intake, and key dietary components may be promising strategies to improve skeletal muscle function and prevent atrophy. Future research should be directed toward personalized nutritional interventions to optimize musculoskeletal health.

